# Shear strength characteristics of muddy interlayers with different water contents and particle size distributions

**DOI:** 10.1371/journal.pone.0349587

**Published:** 2026-06-17

**Authors:** Zumei Zha, Linwei Li, Yiping Wu, Fasheng Miao, Xiqiong Xiang, Dewu Liao, Zhengchao Wu, Yang Xue

**Affiliations:** 1 College of Resources and Environmental Engineering, Key Laboratory of Karst Georesources and Environment (Guizhou University), Ministry of Education, Guiyang, China; 2 Faculty of Engineering, China University of Geosciences, Wuhan, China; 3 Guizhou Geological and Mineral Foundation Engineering Corporation Limited, Guiyang, China; 4 School of Geosciences, Yangtze University, Wuhan, China; Kielce University of Technology: Politechnika Swietokrzyska, POLAND

## Abstract

As a common weak structural plane, muddy interlayers often govern the stability of consequent bedding rock slopes (CBRSs). This study investigated the influence of water content (WC) and particle size distribution (PSD) on the shear strength characteristics of muddy interlayers in CBRSs. A series of CUTS tests was conducted to investigate the strength properties of remolded soil samples for muddy interlayers under WC and PSD conditions, and the maximum information coefficient (MIC) was used to evaluate the effects. The results revealed that under constant PSD, cohesion initially increased, subsequently decreased, and ultimately stabilized with increasing WC, whereas the internal friction angle (φ) consistently reduced. Under constant WC, increasing the proportion of soil particles (2–4 mm) from 0% to 40% reduced cohesion but increased φ. When the PSD changes, the coefficient of curvature (*C*_*c*_) positively affects cohesion but increases φ, whereas increasing the coefficient of uniformity (*C*_*u*_) has the opposite effect. MIC analysis revealed that changes in shear strength parameters due to variations in *C*_*c*_ and *C*_*u*_ were far greater than those caused by WC, highlighting the dominant role of PSD in controlling shear strength. These findings provide valuable insights for assessing the stability of rock slopes with muddy interlayers of similar compositions.

## 1. Introduction

Geological disasters occur frequently in China due to complex geomorphic environments, intense tectonic activities, frequent rainstorms, and human engineering interventions [1,2]. The mountainous regions of Southwest China are particularly susceptible to rock landslides because of their distinctive topography and geological composition. The abruptness and concealment of these landslides often complicate practical prevention efforts. Consequently, addressing rock landslides is crucial for mitigating geological disasters in Southwest China.

Guizhou Province is recognized as one of the regions in China most severely affected by geological disasters and is characterized by widespread sliding on CBRSs. CBRSs are susceptible to sliding failure, particularly those composed of layered Triassic carbonate rocks with muddy interlayers. Such failures are typically initiated by sliding zones that develop within muddy interlayers [3–5]. For example, on July 8, 2020, a large-scale obliquely inclined bedding rockslide occurred in Shiban Village, initiated by an extreme rainstorm [6]. In 2019, a bedding rockslide occurred in Libo County, driven by the combined effects of structural geology and weak interlayer properties [4]. Furthermore, on January 3, 2022, an engineering disturbance triggered a ruinous landslide in a CBRS containing muddy intercalations in the Triassic Guanling Formation [7–8]. These representative cases collectively reveal the primary controlling factors for consequent bedding rock slope instability in Guizhou Province: engineering loading, rainfall infiltration, and weak interlayer characteristics.

The argillized interlayer is a thin layer embedded in rock masses with alternating soft and hard strata. It is a weak structural plane formed under the long-term combined action of interlayer dislocations and water immersion, characterized by low strength, large deformation, weak intergranular bonding, an uneven particle-size distribution, and frequent particle preferred orientation. The argillized interlayer evolves from the over-consolidated, cemented structure of the original interlayer into a pelitic, loose, or oriented structure, with a higher clay content than the original interlayer but lower density and shear strength. Numerous studies indicate that various factors, including WC, PSD, clay content, and wetting‒drying cycles, collectively influence the shear strength of muddy interlayers [9–11]. Specifically, alterations in the WC significantly modify interparticle interaction forces within clays with muddy interlayers [12]. Moreover, increased WC separates clay minerals, forming a mud slurry that fills pores. This process elevates pore water pressure and reduces effective stress [13–15], thereby diminishing the shear strength of weak interlayers [16–18]. For example, the weak interlayer peak, residual, and long-term strength decrease as WC increases. Additionally, changes in WC exert a more pronounced influence on cohesion than on the φ [19–20]. The shear strength of soil is fundamentally determined by its WC, and the shear strength parameters (i.e., cohesion and internal friction angle) are related primarily to this state [21–22]. Furthermore, as soils approach saturation, matric suction decreases sharply to zero, significantly influencing shear strength [23–24] and potentially reactivating dormant landslides [17].

The coefficient of curvature (*C*_*c*_) and coefficient of uniformity (*C*_*u*_) constitute critical parameters for characterizing the particle size distribution (PSD), which substantially influences the shear strength [25]. The definitions of *C*_*c*_ and *C*_*u*_ were as follows:


$$\begin{array}{c} Cc\text{=}\frac{\overset{\text{2}}{\underset{30}{d}}}{{d60\times d}_{10}} \end{array}$$ (1)

$$\begin{array}{c} C\text{u}=\frac{d_{60}}{d_{10}} \end{array}$$ (2)

For example, Amirpour et al. [26] demonstrated through experiments on three materials under sixteen distinct PSD conditions that variations in *C*_*u*_ significantly affect shear strength. Wang et al. [27] reported that φ increases with the median particle size but decreases with increasing *C*_*u*_. Similarly, Gao et al. [28] established the significant impact of *C*_*u*_ on soil strength. Furthermore, soil particle morphology profoundly influences soil strength [29–31]. Within typical sliding shear ranges, minor alterations in PSD conditions significantly affect the residual φ [32,33].

Many scholars have also studied soil shear strength under combined WC and PSD variation. Bouri et al. [34] conducted shear strength tests at different water and fine particle contents. The results show that water content has a significant effect on shear strength, and both fine silt content and water content significantly affect the mechanical parameters c and φ. Monkul et al. [35] mixed three base sands with different gradations and three non-plastic silts of varying gradations and particle shapes to prepare specimens with different fines contents, and conducted undrained triaxial tests. The results show that the static liquefaction potential of sand increases as the uniformity coefficient of the silt matrix decreases; a reduction in the mean grain diameter ratio, caused by changes in silt gradation, also increases the potential for liquefaction. Mukherjee et al. [36] collected low-liquid-limit silty clay (CL-ML) and prepared five groups of specimens by mixing the original soil with 12.5%, 25%, 50%, 75%, and 87.5% sand, respectively, to investigate variations in cohesion (c), internal friction angle (φ), and other parameters. Wei et al. [37] conducted a series of large-scale direct shear tests with varying water contents and grain-size distributions to examine how water content and particle size affect the mechanical properties of soil-rock mixtures (S-RMs). The research indicates that as water content increases, soil strength gradually decreases.

Clearly, both WC and PSD significantly affect the shear strength of muddy interlayers. However, studies on the shear strength characteristics of muddy interlayers under coupled changes in WC and PSD remain limited. The relative importance of these two factors in controlling shear-strength variation remains unclear and warrants further in-depth study. Therefore, this study focuses on the combined influence of WC and PSD on the shear behavior of muddy interlayers and examines which factor plays a more dominant role.

The maximum information coefficient (MIC) was first proposed by Reshef et al. in Science in 2011. As a nonparametric statistical method based on mutual information, MIC quantifies the strength of correlation between variables by dynamically dividing a grid [38]. Unlike the traditional Pearson correlation coefficient, which captures only linear relationships, MIC has strong generality and equitability. It can identify various functional relationships (e.g., exponential, periodic, parabolic) and non-functional relationships, and gives similar scores for different types of correlations with the same noise level, enabling fair comparison [38]. In addition, MIC has strong anti-noise properties and can effectively capture nonlinear coupling relationships even at low signal-to-noise ratios [39]. It is model-free and does not depend on specific distribution assumptions, so it can be well integrated into machine learning algorithms for feature selection. Its value ranges from 0 to 1, which is convenient for setting a unified threshold to screen key influencing factors [39]. Therefore, MIC can unbiasedly identify the complex influence mechanism of soil strength and provide an effective feature selection tool for establishing a reliable triaxial shear strength prediction model, which is difficult to achieve with traditional linear correlation methods.

In summary, this study conducted comprehensive research on remolded samples of muddy interlayers from a landslide in Guizhou Province. A series of consolidated-undrained triaxial shear (CUTS) tests was performed, varying the addition of soil particle content (SPC) with a 2–4 mm diameter and WC. These tests aim to elucidate the shear-strength characteristics and parameters of muddy interlayers from the Triassic Songzikan Formation under varying WC and PSD conditions. Maximum information coefficient (MIC) analysis was employed to examine the interdependence among WC, PSD, cohesion, and φ. The results identify key factors governing the shear strength of muddy interlayers, providing insights beneficial for evaluating slope stability.

## 2. Materials and methods

### 2.1. Overview of sampling location

**Fig 1:**
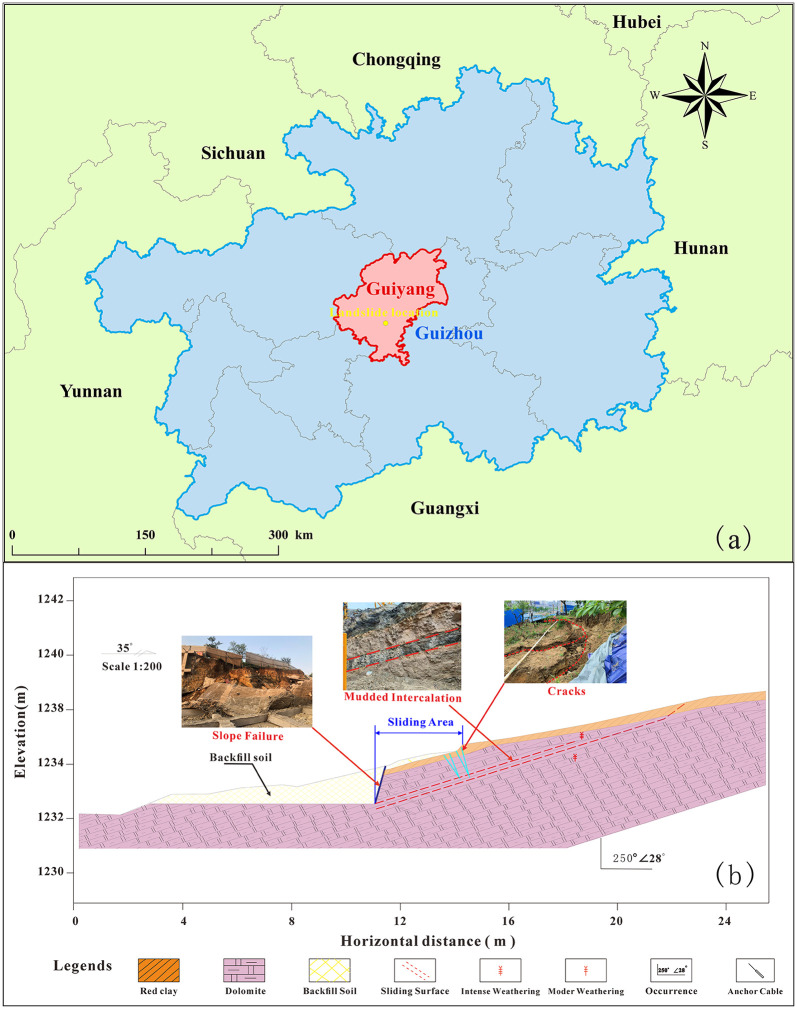
Sampling location of muddy interlayer and its sectional views: (a) Sampling location (base map from Natural Earth); (b) Cross-section.

Fig 1 shows the locations of the muddy interlayer samples and the site’s stratigraphic setting. Regional tectonic faults do not intersect the area. The outcrop consists of argillaceous dolomite of the Triassic Songzikan Formation (T₂sz), with bedding oriented 265°∠28°. Two sets of joint fractures are observed, oriented 201°∠77° and 119°∠65°.

On May 18, 2023, a CBRS at the location experienced sudden, severe deformation and sliding failure during excavation and support work. As illustrated in Fig 1 (b), the landslide boundary was defined by the exposed slope foot and two sets of structural fractures. The weak, muddy interlayers at the base acted as the sliding surface. Several cracks, approximately 1–2 m wide, developed at the trailing edge of the landslide. The terrain exhibits a polyline profile, with a bedrock orientation of 250°∠28° and a slope gradient of approximately 20°. The landslide initiated at the slope front, where the excavation had a slope ratio of 1:0.2 and a height of 8.5 m before failure.

After the landslide, significant deformation occurred along the boundary and within the surrounding rock mass. Tensile cracks formed approximately 10–12 m behind the excavated area, with depths of 2.5–4.8 m and widths of 1–2 m, extending down to the muddy interlayers. Initial field investigations confirmed that the principal sliding surface was the plane of the muddy interlayers. Therefore, the soil samples used in this study were collected from the primary sliding surface (i.e., the muddy interlayers).

### 2.2. Sampling and basic physical characteristics of undisturbed muddy interlayers

The cutting rings, whose inner walls were coated with thin petrolatum, were pressed vertically into the soil during soil sampling. After that, the soil was cut along the exterior of the rings until it protruded above the rings. The collected undisturbed soil samples were first subjected to basic geotechnical tests in accordance with relevant standards to determine their fundamental physical properties. The WC was measured using the oven drying method. Specifically, soil samples were dried at 105 °C for 24 hours to assess natural WC, and saturated WC was also measured. The measurements were repeated three times, and the average value was calculated. The liquid and plastic limits were determined using the combined liquid-plastic limit method, with three replicate measurements per sample to obtain average values. The density was determined by the ring knife method, and the mean value was calculated from three replicate samples. All test results were obtained from undisturbed samples. The results are presented in Table 1. After drying, impurities such as gravel were removed from the samples, and the soil agglomerates were disintegrated into individual particles via a rubber hammer and a crusher. Two representative subsamples (each weighing 2.5 kg) were selected for PSD testing. The test was conducted by sieving through a series of standard sieves with distinct aperture sizes (i.e., 4 mm, 1 mm, 0.5 mm, 0.3 mm, 0.2 mm, 0.125 mm, and 0.075 mm), and the results are shown in Table 2.

**Table 1:** Physical indicators of undisturbed soil samples.

Soil sample	Dry density g/cm^3^	Natural WC (%)	Saturated WC (%)	Liquid limit (%)	Plastic limit (%)	Plasticity index
Muddy interlayers	1.69	13.00	18.00	37.02	20.00	17.02

**Table 2:** Particle composition of undisturbed soil samples.

Particle size (mm)	Content (%)	Grain group name	Content (%)
4−1	7.1	Sand	92
1-0.5	21		
0.5-0.3	16.3		
0.3-0.2	35.3		
0.2-0.125	6		
0.125-0.075	6.3		
<0.075	8	Fine-grained	8

**Fig 2:**
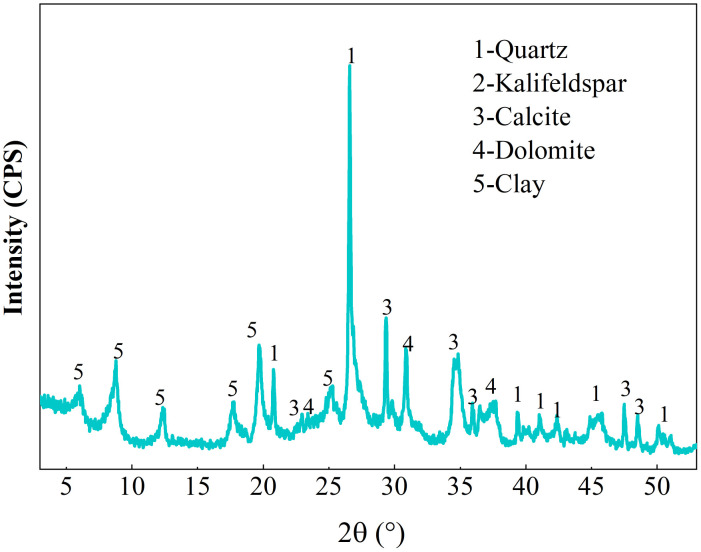
X-ray diffraction pattern of muddy interlayer.

In addition, the mineral composition of undisturbed soil samples was determined using a RAGIKU IV X-ray diffractometer (XRD). XRD analysis was performed on undisturbed soil samples from the weakly argillized interlayer, and the corresponding XRD results are presented in Tables 3 and 4. The proportion of clay minerals in the undisturbed soil samples was 51.2%, indicating that the degree of argillization of the interlayer was high. Moreover, the illite content was 48.4%, followed by the illite/smectite mixed layer, while the contents of kaolinite and chlorite were relatively low. A single XRD measurement was performed. The quantitative assessment of individual mineral phases was conducted using the quantitative standard SY/T5163-2010 [40]. The 2θ range of the XRD pattern for the muddy interlayer is shown in Fig 2.

**Table 3:** Mineral composition of undisturbed soil samples.

Total clay mineral content (%)	Quartz (%)	Kalifeldspar (%)	Calcite (%)	Dolomite (%)
51.2	23	5.3	11.6	8.9

**Table 4:** Composition of clay minerals in undisturbed soil samples.

Clay mineral content (%)						Illite/smectite mixed layer ratio (%)		Green/Mixed Layer Ratio (%)	
Montmorillonite	Illite	kaolinite	Chlorite	Illite/smectite mixed layer	Green/muddy layer	Montmorillonite layer	Illite layer	Montmorillonite layer	Green mudstone layer
0	48.4	2.4	9.3	35.2	4.7	35	65	50	50

### 2.3. Sample preparation and test methods

#### 2.3.1. Experimental scheme.

In traditional CUTS tests with a sample size of 39.1 mm × 80 mm, soil particles larger than 2 mm are typically removed to reduce the size effect. However, in this study, a small number of large 2–4 mm soil particles were present in the undisturbed samples of the muddy interlayers, as shown in Table 2. Therefore, based on the standard sample preparation specification (i.e., maximum particle size ≤ 1/5 of the sample diameter), an experimental scheme with different gradations was designed to examine how residual 2–4 mm particles affect the mechanical response of the muddy interlayers. The original gradation used in the study is shown in Table 2, and the basic physical properties of the soil are presented in Table 1. Notably, the content of 1–4 mm particles in the original soil structure was 7.1%, close to 10%. Therefore, a 10% gradient was adopted for this particle fraction in the test scheme, while a 1.67% gradient was used for WC. This study explored the combined effects of WC (i.e., 13.00%, 14.67%, 16.33%, and 18.00%) and different gradation schemes on the shear strength of remolded soil samples for muddy interlayers. There were 80 test conditions, and the detailed test scheme is shown in Table 5.

**Table 5:** Gradation scheme.

Serial Number	Content of different soil particle sizes (%)								*C*_*c*_	*C*_*u*_
	2-4 mm	1-2 mm	0.45-1 mm	0.3-0.45 mm	0.2-0.3 mm	0.125-0.2 mm	0.075-0.125 mm	<0.075 mm		
1	0	7.1	21	16.3	35.3	6	6.3	8.0	1.58	3.91
2	10	6.4	18.9	14.7	31.8	5.4	5.7	7.2	1.22	4.20
3	20	5.7	16.8	13.1	28.2	4.8	5.1	6.4	0.89	4.93
4	30	4.9	14.7	11.4	24.7	4.2	4.4	5.6	0.25	6.73
5	40	4.2	12.6	9.8	21.2	3.6	3.8	4.8	0.26	7.02

#### 2.3.2. Sample preparation.

**Fig 3:**
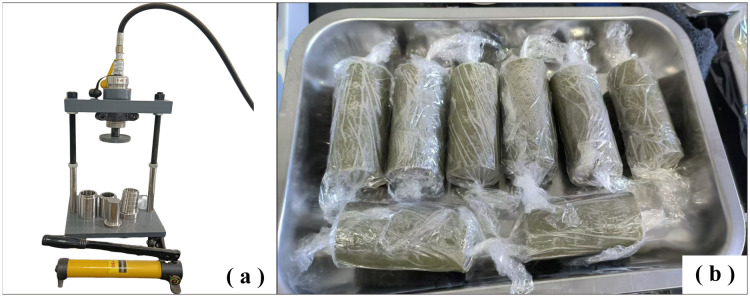
Static compaction of remolded soil samples: (a) Static compaction equipment; (b) Remolded soil samples.

In this study, remolded soil samples of muddy interlayers were prepared using the static compaction method. The equipment used is shown in Fig 3. The procedure was as follows: (1) Calculate the required water addition based on the target moisture content; (2) Apply water via atomized spraying to ensure uniform moisture distribution; (3) Seal the prepared soil samples and allow them to stand for 24 h to ensure adequate water infiltration and homogeneous distribution. Strict control of ambient temperature and humidity was maintained throughout the preparation process to prevent moisture evaporation and ensure testing accuracy. The water content of each batch was verified before sample formation. Before static compaction, the inner wall of the triaxial mold was lubricated with petrolatum to prevent adhesion. The preweighed soil was divided into five layers and compacted layer by layer in a static compaction device. Before placing a new layer, the compacted layer was scarified to improve interlayer bonding and sample integrity. All specimens used in this test were fully remolded.

Saturation was performed via vacuum for remolded samples with a target saturation water content of 18.00%. The setup included a vacuum pump, a saturation cylinder, and a gauge. After sample installation, the vacuum valve was opened, and the water valve was closed, allowing the sample to sit under a pressure of −0.1 MPa for at least 0.5 hours. Deaired water was then introduced to submerge the sample, atmospheric pressure was restored, and the sample was allowed to saturate for 24 hours. Prior to testing, the B-value was measured and confirmed to exceed 0.95.

#### 2.3.3 Test methods.

2.3.3.1 CUTS test: CUTS tests were conducted using a KTL-DTS apparatus produced by Xi’an Kangtuoli Instrument Equipment Co., Ltd., China, with specifications including a maximum axial force of 10 kN, a frequency of 5 Hz, and an axial stress test accuracy of ≥0.1 kPa. The four parallel samples were subjected to CUTS tests at four confining pressures (100, 200, 300, and 400 kPa) under specific WC and PSD conditions. During the consolidation stage, the sample’s volume change was controlled to remain below 5 mm³ over 10 minutes. In the shearing stage, equal-strain shearing was adopted. Typically, the strain rate for clay in shear tests ranges from 0.04% to 0.1% per minute. Thus, in this study, a shear strain rate of 0.0625% per minute was used for the CUTS tests. Given a specimen height of 80 mm, this corresponds to a loading rate of 0.05 mm/min. The maximum axial strain during the shearing process was set to 15%. If no peak was observed in the deviatoric stress‒strain curve, the deviatoric stress at an axial strain of 15% was taken as the peak shear strength of the soil samples. All test data were collected via an automated data-acquisition and processing system that supported the testing instruments. The shear strength parameters of the remolded samples, i.e., cohesion and φ, were calculated according to the Mohr‒Coulomb law.

2.3.3.2 Maximum information coefficient method: The MIC is a statistical measure that quantifies the degree of dependence between two variables [38]. It provides a consistent measure across linear, nonlinear, and periodic associations. Compared with the current distance correlation, Spearman’s correlation coefficient, and methods based on principal curves, the MIC demonstrates relatively excellent metrological performance. The MIC is based on the concept of mutual information (MI). It aims to find an optimal two-dimensional histogram or grid that maximizes the mutual information between the two variables. MI measures the degree of dependence between two random variables. When two variables are completely independent, the MI is zero. Conversely, the MI reaches its maximum when two variables are entirely dependent. On the basis of this principle, the calculation formula for the MIC can be expressed as follows:


$$\begin{array}{c} MIC\text{=}\frac{maxi,jI\left(Xi,Yj\right)}{H\left(X\right)} \end{array}$$ (3)

where I(Xi,Yj) is the MI between *X* and *Y* under the grid division of *X* and *Y* into i × j. H(X) is the entropy of *X*. The closer the MIC value is to 1, the stronger the relationship between the two variables is, whether linear, nonlinear, or complex periodic. Conversely, the two variables are almost independent if the MIC is close to 0. The MIC provides a general method for evaluating data relationships by maximizing the MI and standardizing the results. It is applicable to continuous, discrete, and mixed data types. In this study, the MIC was used to analyze the strength of the dependence between the WC, *C*_*c*_, and *C*_*u*_ and shear strength indices.

## 3. Shear strength characteristics of remolded soil samples for muddy interlayers

### 3.1. Deviatoric stress‒strain curves

**Fig 4:**
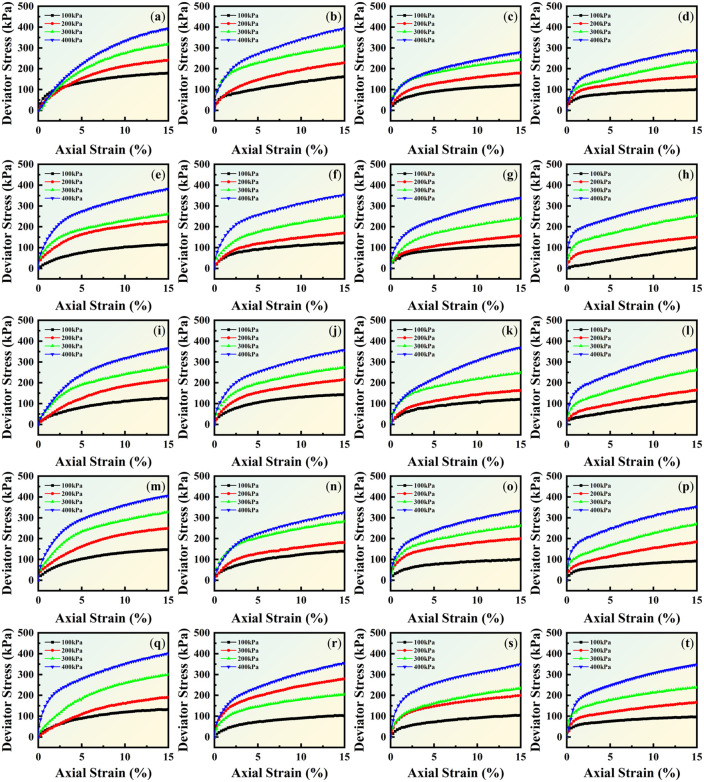
Deviatoric stress‒strain curves of remolded soil samples under different WC and PSD conditions: (a)–(d) 2-4 mm SPC = 0% with WC = 13.00%, 14.67%, 16.33%, and 18.00%; (e)–(h) 2-4 mm SPC = 10% with WC = 13.00%, 14.67%, 16.33%, and 18.00%; (i)–(l) 2-4 mm SPC = 20% with WC = 13.00%, 14.67%, 16.33%, and 18.00%; (m)–(p) 2-4 mm SPC = 30% with WC = 13.00%, 14.67%, 16.33%, and 18.00%; (q)–(t) 2-4 mm SPC = 40% with WC = 13.00%, 14.67%, 16.33%, and 18.00%.

The deviatoric stress‒strain curves of the remolded soil samples for muddy interlayers under different WC and PSD conditions are shown in Fig 4. As the confining pressure increases, the maximum deviatoric stress also increases, indicating that, at a constant WC and PSD, higher confining pressure increases strength. All the deviatoric stress‒strain curves exhibited strain hardening with increasing shear stress; thus, no discernible peaks were observed. Consequently, the deviatoric stress at an axial strain of 15% was taken as the shear strength of the soil samples, with specific values summarized in Table 6.

**Table 6:** Peak shear strength of remolded soil samples under different WC and PSD conditions.

2-4 mm SPC (%)	WC (%)	100 (kPa)	200(kPa)	300 (kPa)	400 (kPa)
0	13.00	180.40	241.14	316.12	393.48
	14.67	162.40	228.84	307.83	394.58
	16.33	122.08	180.27	242.58	280.11
	18.00	101.19	161.99	233.25	290.28
10	13.00	115.47	226.81	319.37	380.94
	14.67	124.55	169.49	250.81	352.90
	16.33	113.67	156.19	260.07	339.28
	18.00	100.03	150.42	252.96	341.10
20	13.00	126.99	213.73	274.08	366.11
	14.67	143.99	215.01	272.83	356.95
	16.33	121.72	162.59	248.11	368.44
	18.00	112.89	165.08	262.02	358.15
30	13.00	148.08	250.20	326.38	405.98
	14.67	140.87	181.69	281.78	325.33
	16.33	100.84	200.10	261.24	335.90
	18.00	92.41	183.24	269.64	353.85
40	13.00	133.67	191.36	299.10	402.63
	14.67	102.18	204.27	273.56	360.95
	16.33	104.94	204.81	233.54	338.64
	18.00	97.78	166.80	237.09	350.69

### 3.2. Changes in the shear strength with varying WC or PSD

According to ASTM D4767 [41], during undrained shear, pore-water pressure shall be measured in a manner that minimizes water flow into or out of the specimen. To meet this requirement, high-stiffness electronic pressure transducers or null-indicating devices must be used. Because conventional research equipment often fails to satisfy this condition, resulting in limited reliability in pore-pressure measurement, this study uses consolidated undrained (CU) triaxial tests to determine soil shear strength parameters, with the objective of establishing a strength parameter system required for total-stress analysis.

**Fig 5:**
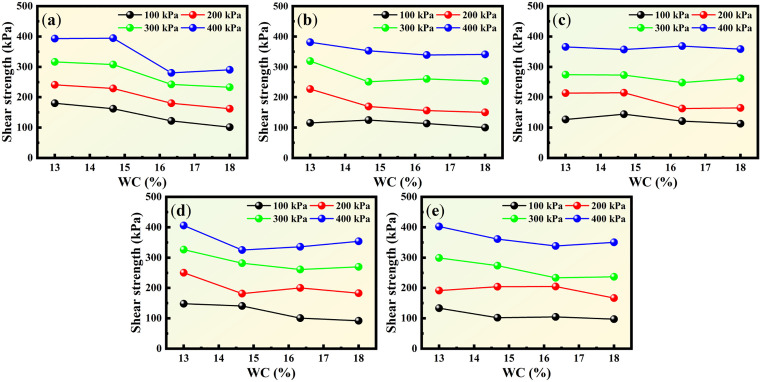
Shear strength of remolded samples for muddy interlayers with same 2–4 mm SPC: (a) 0%; (b) 10%; (c) 20%; (d) 30%; (e) 40%.

Fig 5 shows how the shear strength of remolded soil samples with muddy interlayers changes with increasing WC under identical PSD conditions. When the content of soil particles with diameters between 2 mm and 4 mm remained constant, the shear strength of remolded samples from muddy interlayers decreased with increasing WC at identical confining pressures. As the WC increased further, the rate of decrease in shear strength for the same soil particle content between 2 mm and 4 mm slowed noticeably. For example, when the WC increased from 16.33% to 18.00%, the average decrease in shear strength was slightly lower than that from 13.00% to 16.33%.

**Fig 6:**
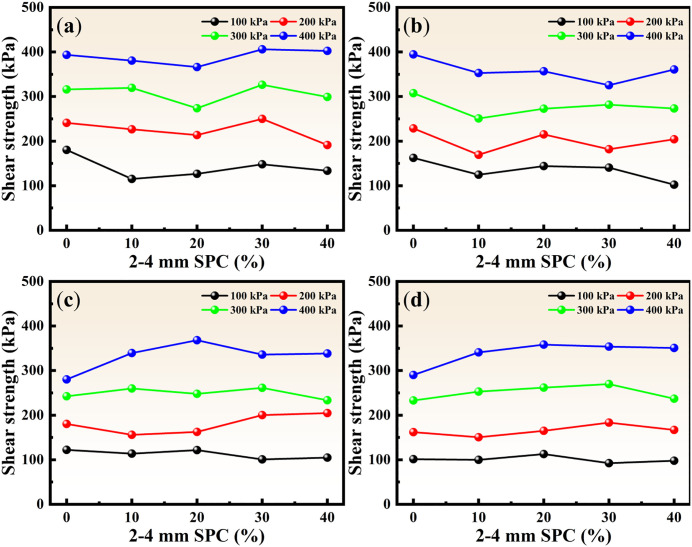
Shear strength of remolded samples for muddy interlayers with same WC: (a) 13.00%; (b) 14.67%; (c) 16.33%; (d) 18.00%.

Fig 6 shows how shear strength varies with PSDs under identical WCs. When the proportion of soil particles with diameters of 2 mm to 4 mm increased from 0% to 40%, the shear strength decreased at a low confining pressure (i.e., 100 kPa) under the same WC conditions. However, in most cases, when the confining pressure exceeded 100 kPa, the shear strength increased at identical WCs. For example, the shear strength of remolded samples at a confining pressure of 400 kPa increased significantly when the WC was 16.33% or 18.00%.

### 3.3. Variations in shear strength parameters with varying WC and PSD

**Fig 7:**
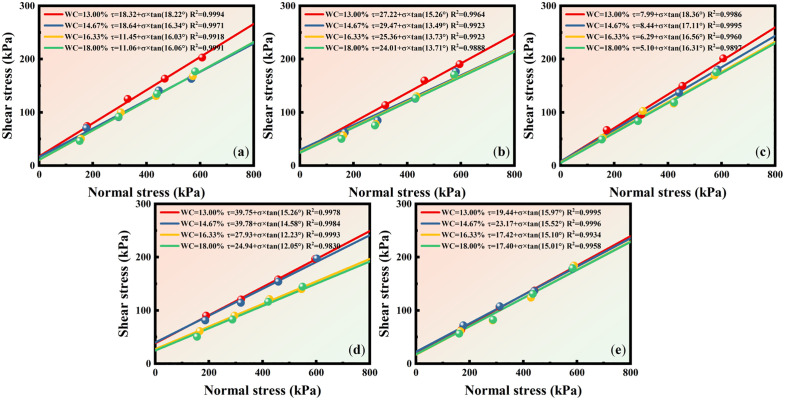
Linear fitting results of remolded soil samples under different WC and PSD conditions: (a) 2-4 mm SPC of 0%; (b) 2-4 mm SPC of 10%;(c) 2-4 mm SPC of 20%; (d) 2-4 mm SPC of 30%; (e) 2-4 mm SPC of 40%.

To adapt to different soil types and general experimental conditions, Cullen proposed the strength theory of soils, whose expression is defined as follows:


$$\begin{array}{c} \tau \text{=}\sigma tan\varphi +c \end{array}$$ (4)

where σ represents the normal stress, φ denotes the φ, and c represents cohesion. There is a linear relationship between τ and σ. Thus, the formula for calculating the φ and cohesion is as follows:


$$\begin{array}{c} \begin{array}{c} \varphi \text{=}arcsin\left(tan\alpha \right)\\ c=\frac{d}{cos\varphi } \end{array} \end{array}$$ (5)

where d is the intercept of the linear fitting function (5). α is the slope of the linear fitting function (5). The shear strength parameters can be determined by performing a linear fit to the CUTS test results. As shown in Fig 7, the fitting results for the shear strength parameters under varying WCs and soil particle contents with diameters between 2 mm and 4 mm showed a high degree of linear fit, with an average R^2^ of 0.9882.

#### 3.3.1 Relationships between shear strength parameters and WC.

**Fig 8:**
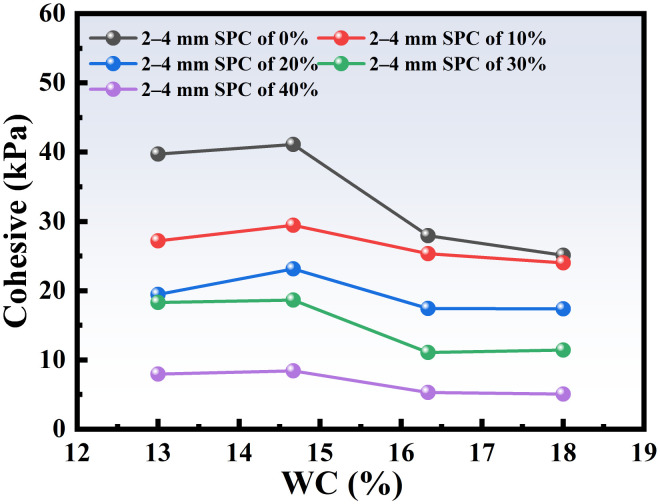
Relationships between WC and cohesion for different 2–4 mm SPCs.

Fig 8 illustrates how cohesion varies with WC under identical PSD conditions. Cohesion initially increased, then decreased, and eventually stabilized as WC decreased. Cohesion reaches its maximum value at a WC of 14.67%. The cohesion ranged from 41.11 kPa to 5.10 kPa. Specifically, when WC was below 14.67%, cohesion increased with increasing WC. However, as WC exceeded 14.67%, cohesion gradually decreased to a relatively stable value.

In addition, under the same WC conditions, the amount of cohesion loss increased significantly as the proportion of soil particles with diameters between 2 mm and 4 mm increased. For example, when the WC was 13.00%, and the content of soil particles with a diameter between 2 mm and 4 mm increased from 0% to 40%, the cohesion decreased from 39.75 kPa to 7.99 kPa. The decrease rate was 84.1%. However, when the WC was 18.00%, the cohesion decreased from 29.94 kPa to 5.10 kPa, a decrease of 83.0%.

**Fig 9:**
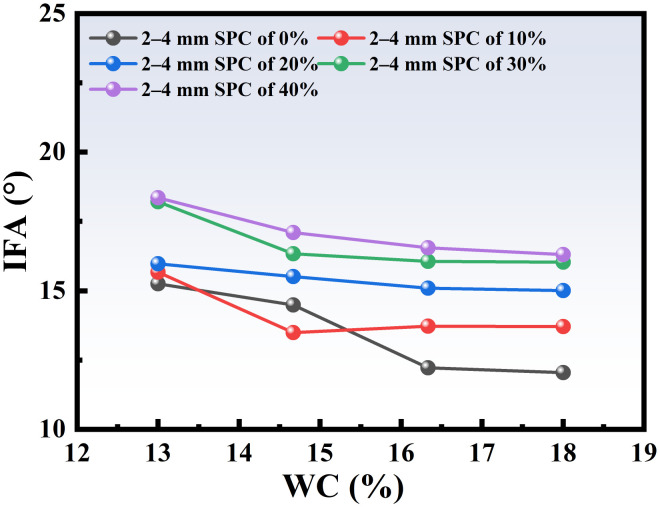
Relationships between the WC andΦ for different 2–4 mm SPCs.

Fig 9 shows how φ varies with WC for identical PSDs. As WC increased, φ generally decreased. Specifically, when the WC was below 14.67%, φ decreased rapidly with increasing WC. After that, the decline in φ tended to stabilize. In most cases, at the same WC, increasing the content of soil particles with diameters between 2 mm and 4 mm significantly increased φ. For example, when the WC was 13.00%, and the content of 2–4 mm soil particles rose from 0% to 40%, the φ increased from 15.26° to 18.36°, an increase of 20.3%. Moreover, when the WC was 18.00%, the φ increased from 12.05° to 16.31°, a 35.4% increase.

#### 3.3.2. Relationships among shear strength parameters, *C*_*c*_, and *C*_*u*_.

As the content of soil particles with diameters between 2 mm and 4 mm increased, the PSD was strongly influenced. However, using a single index (i.e., the 2–4 mm SPC) was inadequate to elucidate the influence of PSD changes on shear strength parameters. *C*_*c*_ and *C*_*u*_, which reflect the uniformity and continuity of the PSD, significantly influence the soil’s mechanical properties. Therefore, *C*_*c*_ and *C*_*u*_ were employed in this study to explore the relationships between shear strength parameters and PSD conditions.

**Fig 10:**
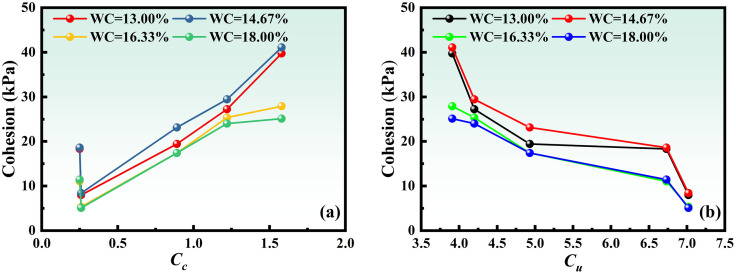
Relationships among *C*_*c*_, *C*_*u*_, and cohesion: (a) Relationships between *C*_*c*_ and cohesion; (b) Relationships between *C*_*u*_ and cohesion.

Fig 10 shows how cohesion varies with *C*_*c*_ and *C*_*u*_ at identical WCs. When the WC remained constant, cohesion increased with *C*_*c*_, indicating a positive correlation between them. Conversely, as the amount of *C*_*u*_ increased gradually, cohesion decreased, indicating a negative correlation between cohesion and *C*_*u*_.

**Fig 11:**
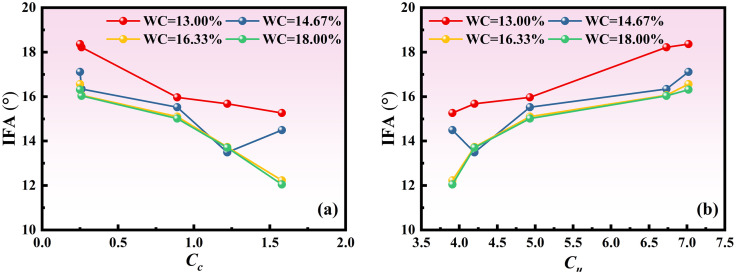
Relationships among *C*_*c*_, *C*_*u*_, andΦ: (a) Relationships between *C*_*c*_ and Φ; (b) Relationships between *C*_*u*_ and Φ.

Fig 11 shows how φ varies with *C*_*c*_ and *C*_*u*_ under identical WC conditions. Under the same WC conditions, the φ gradually decreases as *C*_*c*_ increases, indicating a negative correlation between *C*_*c*_ and the φ. Conversely, when the *C*_*u*_ gradually increased, the φ gradually increased, suggesting a positive correlation between the *C*_*u*_ and the φ.

### 3.4. MICs between shear strength parameters, *C*_*c*_, *C*_*u*_, and WC

**Fig 12:**
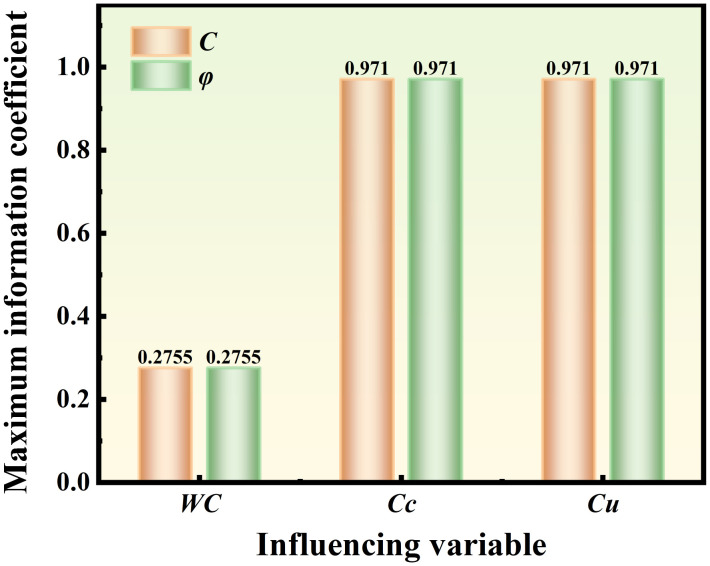
Maximum information coefficient.

This study used the MIC method to quantify relationships among cohesion (*c*), internal friction angle (φ), *C*_*c*_, *C*_*u*_, and WC. Fig 12 shows the MICs among the shear strength parameters *C*_*c*_, *C*_*u*_, and WC. 1) MIC(c, WC) = MIC(φ, WC) = 0.2755; 2) MIC(c, *C*_*c*_) = MIC(φ, *C*_*c*_) = 0.9710; 3) MIC(c, Cu) = MIC(φ, *C*_*u*_) = 0.9710. Ordering the MICs from smallest to largest shows that MIC(WC) <<MIC(*C*_*c*_) == MIC(*C*_*u*_). These findings showed that the PSD parameters *C*_*c*_ and *C*_*u*_ had a much stronger relationship with the *c* and φ of mudded intercalations than those of the WC conditions.

## 4. Discussions

### 4.1. Strain hardening characteristics of remolded samples

The fundamental reason for the strain hardening of triaxial remolded samples in the CUTS tests is the complete destruction of the natural structure during sample preparation (drying, crushing, and recompaction), during which cementation and fabric memory are lost. Consequently, the samples can support loads only through continuous shear contraction (volume shrinkage) and particle rearrangement. During shearing, porosity decreases continuously, and density and frictional strength increase synchronously, resulting in a monotonic rise in deviatoric stress with axial strain, without an obvious peak. Li et al. [42] demonstrated through consolidated undrained triaxial tests that remolded samples undergo continuous shear contraction throughout the shearing stage, with pore water pressure increasing steadily, which corresponds to macroscopic continuous hardening. A study on remolded Wenzhou marine silty soil published by Shi [43] further showed that when the external load exceeds the skeleton yield limit, particle reorganization occurs, leading to strain hardening.

### 4.2. Impact of WC variation on shear strength parameters

Cohesion arises from the combined effects of attractive and repulsive forces between soil particles. As shown in Fig 8, the cohesion of remolded samples with distinct PSDs initially increased, then decreased, and ultimately stabilized as WC increased. The peak cohesion consistently occurred at a WC of 14.67%. This behavior can be attributed to the adsorption process as follows. When WC was below the optimum value (approximately 14.67% in this study), increasing WC led to the gradual adsorption of more weakly bound water around the strongly bound water, resulting in a thicker bound water film. This enhanced interparticle attraction manifests macroscopically as increased cohesion. Conversely, when WC exceeded the optimum value, the thicker bound water film increased the interparticle distance, thereby reducing the attractive forces. Consequently, cohesion decreased with increasing WC.

The frictional properties of soils are characterized by φ, which comprises sliding and occlusive friction. Sliding friction arises from particle surface roughness, whereas occlusive friction results from particle embedding, interlocking, and detachment during movement. Fig 9 shows that φ decreased consistently with increasing WC for remolded samples across different PSDs. This reduction occurred because increasing WC altered the thickness and compactness of the water film bound to particle surfaces. A thicker water film diminished interparticle biting forces. As a sliding interface between particles, the water film directly contributed to the reduction in φ. This reduction exhibited limiting behavior, as evidenced by a decreasing rate of change with increasing WC.

Overall, the relationships among WC, cohesion, and φ observed in this study align with findings reported by Wei et al. [44]. However, a notable observation is that samples with favorable gradation showed significantly less sensitivity of cohesion and φ to WC variation than poorly graded samples. Specifically, when the 2–4 mm SPC was 20%, the *C*_*c*_ was 0.89, and the *C*_*u*_ was 4.93. This PSD condition, markedly closer to a well-graded state than the other four tested conditions, exhibited the smallest decreases in cohesion and φ with increasing WC (as shown in Figs 8 and 9). The final reductions were only 0.96 kPa for cohesion and 2.04° for φ, significantly lower than those observed under other PSD conditions. This reduced sensitivity is attributed to the broad, continuous particle-size distribution inherent in well-graded soils. In these soils, fine particles effectively fill the voids left by coarser particles, thereby enhancing structural integrity and stability. This robust structure makes the soil less susceptible to WC variation.

### 4.3. Impact of variations in *C*_*c*_ and *C*_*u*_ on shear strength parameters

Under constant WC, Fig 6 shows shear strength variation with 2–4 mm particles. At low WC (13.00%), strength drops at 10% coarse particles, then recovers, peaking at 30–40%. As in Zhang et al. [45], low coarse content prevents particles from forming stable force chains, thereby lowering φ. With increased water (14.67–16.33%, Figs 6b–c), the optimal coarse content shifts to 20–30%, with more consistent strength and earlier stabilization. Dai et al. [46] noted that binary granular mixtures show PSD-dependent shear strength, with optimal gradation maximizing strength. Intermediate coarse fractions improve interlocking [47]. At high water (18.00%, Fig 6d), strength stabilizes across the range, with an optimum at 20–30%. Sensitivity decreases as pore-water pressure effects outweigh particle interlocking, approaching clay-like behavior [48]. All curves converge at 20–30%, indicating a weakening of the skeleton’s influence as lubrication increases.

*C*_*c*_ quantifies the curvature of the PSD curve. Elevated *C*_*c*_ values (i.e., *C*_*c*_ > 3) or extremely low values (i.e., *C*_*c*_ < 1) indicate a deficiency of particles within specific size ranges. Typically, a *C*_*c*_ ranging from 1 to 3 is considered a necessary and sufficient condition for well-graded soils. As shown in Fig 10(a), cohesion increased progressively as *C*_*c*_ rose from 0.25 to 1.58. This enhancement was attributable to the soil gradation evolving toward optimal conditions during this interval, characterized by a more continuous PSD. Consequently, the interparticle contact area and the number of contact points increased, leading to higher cohesion.

Notably, however, a marginal increase in *C*_*c*_ from 0.25 to 0.26 resulted in a marked decrease in cohesion. For the soil sample with *C*_*c*_ = 0.25, the *C*_*u*_ was 6.73, whereas at *C*_*c*_ = 0.26, the *C*_*u*_ was 7.02. Although the change in *C*_*c*_ was minimal, the difference in *C*_*u*_ was relatively significant. The soil samples with *C*_*c*_ = 0.25 and *C*_*u=*_6.73 exhibited a smaller disparity between large and small particles than those with *C*_*c*_ = 0.26 and *C*_*u=*_7.02. This configuration substantially increased the interparticle contact area and the number of contact points, thereby enhancing cohesion at *C*_*c*_ = 0.25.

As illustrated in Fig 11(a), φ decreased progressively as *C*_*c*_ increased from 0.25 to 1.58. This reduction occurred because, during this process, the proportion of relatively strong particles (diameters between 2 mm and 4 mm) gradually decreased, and the size disparity between large and small particles progressively diminished. Consequently, particle surface roughness decreased, and the mechanical interlocking effect between soil particles became less pronounced. Therefore, the friction force decreases with increasing *C*_*c*_.

*C*_*u*_ reflects the degree of nonuniformity within the soil PSD. A larger *C*_*u*_ indicates a more nonuniform PSD and a greater disparity between coarse and fine particles. In this study, *C*_*u*_ increased significantly as the SPC diameter increased from 2 mm to 4 mm. This compositional shift increased the proportion of large particles while decreasing the proportion of small particles. This change significantly reduced the interparticle contact area and the number of contact points, resulting in a gradual decrease in cohesion, as shown in Fig 10(b).

As shown in Fig 11(b), cohesion decreased continuously with increasing *C*_*u*_, whereas the φ of the remolded sample with muddy interlayers increased progressively. This phenomenon occurred because incorporating more particles between 2 and 4 mm increases the quantity of high-strength coarse particles and increases their surface roughness. This enhanced roughness facilitates mechanical interlocking between particles during shearing, increasing the friction force as *C*_*u*_ increases. These findings agree with those of Lim et al. [49], further corroborating that soil gradation significantly influences peak strength and φ, and that an increase in *C*_*u*_ elevates the soil’s φ.

More in depth, as the coarse particle content increases gradually, the engineering properties of the weak interlayer transition from matrix-controlled to skeleton-controlled. The friction coefficient increases monotonically with coarse particle content, while cohesion decreases due to a lower density of the effective clay phase. After coarse particles form a stable skeleton, the shear band is forced to pass through particle contact points, resulting in significant dilatancy and interlocking reinforcement. An increase in coarse particle content reduces the shear surface’s fractal dimension and increases its surface roughness; meanwhile, the particle breakage rate shows an exponential positive correlation with coarse particle content, and the energy dissipation induced by particle breakage further amplifies the apparent friction. These microscopic changes are manifested macroscopically as an increase in φ, whereas a reduction in the fine-grained cementation phase weakens cohesion.

### 4.4. Comparative influence of WC and PSD conditions on shear strength parameters

Based on the data in Table 7, the variation range (i.e., maximum minus minimum) of shear strength parameters during WC increments was quantified under constant PSD conditions. For samples with 0%, 10%, 20%, 30%, and 40% SPC between 2 mm and 4 mm, the cohesion variation ranges were 15.98 kPa, 5.46 kPa, 5.77 kPa, 7.19 kPa, and 3.34 kPa, respectively. The mean cohesion variation was 7.548 kPa. The φ variation ranges were 3.21°, 1.97°, 0.96°, 2.19°, and 2.05°, yielding a mean φ variation of 2.076°.

**Table 7:** Linear fitting results of shear strength parameters for remolded soil samples of muddy interlayers.

2-4 mm SPC (%)	WC (%)	Cohesion (kPa)	Φ (°)
0	13.00	39.75	15.26
	14.67	41.11	14.58
	16.33	27.93	12.23
	18.00	24.94	12.05
10	13.00	27.22	15.68
	14.67	29.47	13.49
	16.33	25.36	13.73
	18.00	24.01	13.71
20	13.00	19.44	15.97
	14.67	23.17	15.52
	16.33	17.42	15.10
	18.00	17.40	15.01
30	13.00	18.32	18.22
	14.67	18.64	16.34
	16.33	11.06	16.06
	18.00	11.45	16.03
40	13.00	7.99	18.36
	14.67	8.44	17.11
	16.33	6.29	16.56
	18.00	5.1	16.31

Conversely, the variation in shear strength parameters under different PSD conditions was analyzed at fixed WC levels. For the samples with WCs of 13%, 14.67%, 16.33%, and 18%, the cohesion variation ranges were 31.76 kPa, 32.67 kPa, 22.61 kPa, and 20.03 kPa, respectively, with a mean of 26.7675 kPa. The measured φ variations were 3.1°, 2.62°, 4.33°, and 4.26°, resulting in a mean φ variation of 3.5775°.

Compared with increasing the water content from 13% to 18%, elevating the 2–4 mm particle fraction in the studied soil from 0% to 40% has a greater effect on soil cohesion and φ. Consistent with the correlation analysis presented in Section 3.4, this finding confirms that PSD conditions exert significantly stronger control over the soil shear strength parameters than WC conditions.

### 4.5 Research limitations

This investigation elucidated the effects of WC and PSD conditions on the shear strength of argillaceous intercalations. However, due to triaxial test specimen size constraints, the maximum particle diameter in this experiment was limited to 4 mm. Field investigations revealed abundant larger-diameter particles, whose influence would require large-scale triaxial testing to determine shear strength parameters. Furthermore, the dataset was derived from a specific region and a particular intercalation type, potentially limiting the generalizability of the conclusions. Research should expand sample sizes and incorporate data from diverse geological settings to increase the representativeness and reliability of the findings.

## 5. Conclusions

The shear strength parameters of muddy interlayers are critical for slope stability analysis. To mitigate CBRS sliding in mountainous regions of Southwest China, particularly in Guizhou Province, precise characterization of the shear strength of the muddy interlayer under varying WC and PSD conditions is imperative. Consequently, a series of CUTS tests was conducted to investigate the strength properties of remolded soil samples from muddy interlayers within the Triassic Songzikan Formation. The effects of WC, *C*_*c*_, and Cu on cohesion and φ were examined using MIC analysis. The following conclusions were drawn:

(1) The CUTS results showed that remolded muddy interlayer soils exhibited strain hardening without a peak strength. Cohesion increased and then stabilized with rising WC, while φ declined continuously; increasing SPC by 2–4 mm raised φ but reduced cohesion.
(2) Increasing *C*_*c*_ enhanced cohesion but reduced φ, whereas increasing *C*_*u*_ raised φ but lowered cohesion. Superior gradations showed less sensitivity of shear strength parameters to WC variation than inferior gradations.
(3) Meticulous analysis of the MICs among the WC, *C*_*c*_, *C*_*u*_, and shear strength parameters (i.e., cohesion and φ), along with the correlation of relative change amplitudes, revealed that the influences of *C*_*c*_ and *C*_*u*_ on cohesion and φ were significantly more pronounced than those of the WC conditions.
(4) Shear strength behavior resulted from the coupled effects of WC and PSD: WC regulated interparticle bonding via bound-water films and clay bridges, while PSD controlled the soil skeleton structure. Gradation parameters dominated because the skeleton exerted greater control over shear strength than particle surface water films.
(5) These findings enhance understanding of shear-strength mechanisms in muddy interlayers and have practical implications for slope stability assessment and prevention in Southwest China. For engineered applications such as soil stabilization or reconstructed fills, optimizing PSD through controlled gradation and coarse-particle content is an effective way to improve shear resistance and ensure long-term stability.

## Supporting information

S1 File Triaxial test data” and it is a compressed archive that contains all the triaxial test data used to generate Figure 4. (RAR)
